# Protein–Protein Interfaces from Cytochrome c Oxidase I Evolve Faster than Nonbinding Surfaces, yet Negative Selection Is the Driving Force

**DOI:** 10.1093/gbe/evu240

**Published:** 2014-10-29

**Authors:** Juan Carlos Aledo, Héctor Valverde, Manuel Ruíz-Camacho, Ian Morilla, Francisco Demetrio López

**Affiliations:** ^1^Departamento de Biología Molecular y Bioquímica, Facultad de Ciencias, Universidad de Málaga, Spain; ^2^Departamento de Estadística e Investigación Operativa, Facultad de Ciencias, Universidad de Málaga, Spain

**Keywords:** cytonuclear coevolution, COX, evolutionary rates, mitochondria, oxidative phosphorylation, protein evolution

## Abstract

Respiratory complexes are encoded by two genomes (mitochondrial DNA [mtDNA] and nuclear DNA [nDNA]). Although the importance of intergenomic coadaptation is acknowledged, the forces and constraints shaping such coevolution are largely unknown. Previous works using cytochrome c oxidase (COX) as a model enzyme have led to the so-called “optimizing interaction” hypothesis. According to this view, mtDNA-encoded residues close to nDNA-encoded residues evolve faster than the rest of positions, favoring the optimization of protein–protein interfaces. Herein, using evolutionary data in combination with structural information of COX, we show that failing to discern the effects of interaction from other structural and functional effects can lead to deceptive conclusions such as the “optimizing hypothesis.” Once spurious factors have been accounted for, data analysis shows that mtDNA-encoded residues engaged in contacts are, in general, more constrained than their noncontact counterparts. Nevertheless, noncontact residues from the surface of COX I subunit are a remarkable exception, being subjected to an exceptionally high purifying selection that may be related to the maintenance of a suitable heme environment. We also report that mtDNA-encoded residues involved in contacts with other mtDNA-encoded subunits are more constrained than mtDNA-encoded residues interacting with nDNA-encoded polypeptides. This differential behavior cannot be explained on the basis of predicted thermodynamic stability, as interactions between mtDNA-encoded subunits contribute more weakly to the complex stability than those interactions between subunits encoded by different genomes. Therefore, the higher conservation observed among mtDNA-encoded residues involved in intragenome interactions is likely due to factors other than structural stability.

## Introduction

Although mitochondria are involved in many aspects of cell function, including proliferation (Aledo 2004), apoptosis (Suen et al. 2008), and aging (Aledo et al. 2011), their central role is related to energy transduction in oxidative phosphorylation (OXPHOS). The mitochondrial proteins responsible for the OXPHOS are encoded by two genomes. In mammals, the mitochondrial genome (mitochondrial DNA [mtDNA]) encodes for 13 polypeptides that interact with a large number of nuclear-encoded (nuclear DNA [nDNA]-encoded) polypeptides to form the functional complexes I, III, IV, and V of the OXPHOS system. Given that each mtDNA gene product must interact with proteins encoded by the nuclear genome to carry out its functions, coevolution between mtDNA and nDNA leading to intergenomic coadaptation is expected.

As a consequence of their critical function, mutations altering the structure of these oligomeric complexes must face the close scrutiny of natural selection. In this way, natural selection will favor evolutionary coadaptation of interacting proteins, either to improve physiological functions (Schmidt et al. 2005) or just to maintain the fitness through compensatory changes after a slightly deleterious mutation has been fixed by genetic drift (Osada and Akashi 2012). Whatever the driving force may be, there are numerous examples of the biological importance of intergenomic coadaptation. In this sense, xenomitochondrial cybrid cells constructed using nDNA from one species and mtDNA from a close species were viable and had a functional OXPHOS, whereas more divergent species failed to produce functional OXPHOS complexes (Kenyon and Moraes 1997; Barrientos et al. 2000; McKenzie et al. 2003). These results underline the importance of cytonuclear coevolution. Similar conclusions were derived from studies where repeated backcrossing of genetically isolated populations of the copepod *Tigriopus californicus* allowed to place the maternally inherited mtDNA genome of one population with the paternal nDNA of another. These interpopulation hybrids exhibited a defective OXPHOS system (Edmands and Ronald 1999). A third line of evidence suggesting that OXPHOS proteins have coevolved to function optimally comes from epistatic studies where mutations in mtDNA genes that are pathogenic in humans have been observed in naturally occurring genomes from nonhuman mammals (de Magalhaes 2005; Azevedo et al. 2009).

Although the above studies emphasize the relevance of coevolution between interacting proteins, they do not provide much insight into the forces and constraints shaping such coevolution. In an attempt to explore the evolutionary dynamics of these protein–protein interactions, Schmidt et al. 2001 used cytochrome c oxidase (COX) as a model of OXPHOS holoenzyme. These authors analyzed the rate of nonsynonymous substitutions within a set formed by mtDNA-encoded residues in physical proximity to nDNA-encoded amino acids, and compared it with that computed for the rest of mtDNA-encoded residues, which are not in contact with nDNA-encoded polypeptide chains. They concluded that mtDNA-encoded residues in close contact with amino acids being encoded by the nucleus evolve faster than the rest of mtDNA-encoded residues (Schmidt et al. 2001). This result was interpreted as being due to many different amino acid replacements among the close contact residues being required to optimize this protein’s interaction with other proteins. Actually, the authors referred to such a state as an “optimizing interaction.” In contrast, when COX residues encoded by nDNA were segregated on the basis of proximity to mtDNA-encoded residues, and the rates of nonsynonymous substitution were analyzed, the conclusion reached was the opposite. That is, those nDNA-encoded residues in contact with mtDNA-encoded amino acids evolve more slowly than the rest of nDNA-encoded residues.

These striking results have been often cited as an example of the differential forces driving mtDNA and nDNA evolution (Das et al. 2004; Willett and Burton 2004; Castellana et al. 2011; Aledo et al. 2012). Although the constrained evolution of nDNA-encoded interacting residues is in line with the prevailing view that protein evolution is generally conservative and constraining interactions are typical, the observation that mtDNA-encoded interacting residues evolve at much higher rates than noninteracting amino acids, if confirmed as a bona fide observation, deserved a sound explanation. Herein, we have revisited the “optimizing interaction” hypothesis. Using a comprehensive number of mammalian taxa and extended statistical analyses, we have found that, in general, interacting mtDNA-encoded residues, alike interacting nDNA-encoded residues, are subjected to higher constraints than their corresponding noninteracting counterparts. We also provide the keys to understand why previous studies failed to reach similar conclusions. More concretely, we describe a remarkable conservation degree of the nonbinding surface of COX I, which went unnoticed in previous studies. Neglecting this exceptional behavior of the noncontact surface of COX I can be misleading. In addition, we show an intriguing difference in the evolutionary rate of mtDNA-encoded residues depending on whether they contact with nDNA-encoded subunits or with other mtDNA-encoded subunits.

## Materials and Methods

### DNA Sequences

A collection of 371 mammalian mitochondrial genomes was obtained from the National Center for Biotechnology Information genome database (www.ncbi.nlm.nih.gov). A complete list of the mammalian taxa used and the accession number of the sequences is provided as supplementary material S1, Supplementary Material online. Orthologous sequences were aligned by codons using ClustalW.

### Codon Sorting

**Fig. 1.—:**
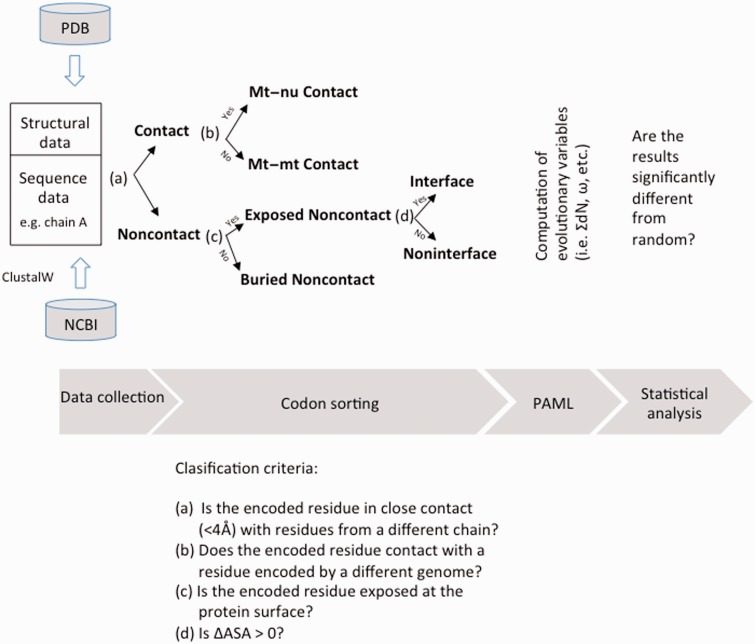
Flowchart for the main methodological procedure adopted. Once sequence and structural data were collected, aligned codons were sorted into different subsets according to the criteria sketched in the figure. Afterwards, the indicated variables were assessed and diverse evolutionary tests were carried out using the package of programs PAML (details given in the text). ASA stands for accessible surface area. For each amino acid from a given subunit, the ASA was assessed in two different ways: 1) In the single subunit, isolated from the rest of the complex, and 2) when the subunit forms part of the complex. Thus, ΔASA = ASA_1_ − ASA_2_ ≥ 0 for any residue. Raw data and a script in R to analyze them are provided as supplementary material S3, Supplementary Material online.

Using the sequence from *Bos taurus* as reference and the crystal structure of bovine COX (Protein Data Bank, PDB, 2OCC), each codon position from the above described alignments was sorted into different subsets according to the algorithm sketched in figure 1. Briefly, the data set corresponding to all the codons from the alignment of a given COX subunit (for instance, chain A, corresponding to COX I, which is a mtDNA-encoded subunit) was initially divided into two subsets: “Contact” and “Noncontact,” depending on whether the encoded amino acid from chain A is or not closer than 4 Å to a residue from any polypeptide other than chain A, respectively. The distance between two amino acids is given by the minimal distance between all pairs of heavy atoms from the two residues. Interacting positions were defined as being less than 4 Å apart because this is the upper limit for weak interactions (Martin et al. 1997). Afterwards, the Contact set was, in turn, split into two subsets: Intergenomic Contact (“Mt–nu Contact,” in the example) and Intragenomic Contact (“Mt–mt Contact,” in the example). The criterion to assign a given codon into the former subset was that the interacting residues should have been encoded by different genomes, otherwise the codon was allocated into the latter subset. On the other hand, the Noncontact set was split up into two subsets: “Exposed Noncontact” and “Buried Noncontact,” on the basis of solvent accessible surface areas of the considered residue (Aledo et al. 2012).

### Phylogenetic Reconstruction

The DNA sequences of the three mtDNA-encoded COX subunits were concatenated for each of the 371 mammalian species analyzed. These concatenated and aligned sequences were used to reconstruct the phylogeny using a method of maximum likelihood implemented in the package PHYLIP (http://evolution.genetics.washington.edu/phylip.html). The obtained tree in Newick format is provided as supplementary material S2, Supplementary Material online.

### Nonsynonymous Sequence Divergences

Nonsynonymous sequence divergences were calculated using two approaches: From pairwise comparisons and on a lineage-by-lineage basis. For the first approach, we used the alignment data subsets described in the precedent section and employed the method described by Yang and Nielsen (2000) to estimate the number of nonsynonymous substitutions per nonsynonymous site. This method is implemented in the package PAML 4.7 (Yang 2007). The sum of these nonsynonymous sequence divergences for all the pairwise comparisons was computed and denoted as Σd*N*. These values were used to compute the so-called interaction ratio, defined, according to Schmidt et al. (2001), as the ratio between the Σd*N* values of the two subsets of residues being compared. For instance, if we want to compare the subsets Mt–nu Contact and Exposed Noncontact, we should compute the interaction ratio Σd*N*_Mt__–__nu_/Σd*N*_ExposedNoncontact._ In this way, values for this ratio indistinguishable from 1 should be interpreted as that both sets of residues show similar nonsynonymous substitution rates. On the other hand, a value below 1 would indicate a reduced evolutionary rate among the residues belonging to the Mt–nu Contact group, relative to those residues from the Exposed Noncontact category. On the contrary, a ratio value over 1 points to a higher rate of nonsynonymous substitutions among Mt–nu Contact residues, with respect to those belonging to the Exposed Noncontact subset.

In order to calculate the number of nonsynonymous substitutions per nonsynonymous site on a lineage-by-lineage basis, we used the reconstructed phylogeny and a maximum-likelihood method (F3×4 model) implemented in codeml from the PAML package (Yang 2007).

### Random Distributions

The statistical support for the conclusion that Exposed Noncontact residues from COX I exhibit a unique behavior, provided in figure 4, was obtained by randomly sampling on the data set of each mtDNA-encoded subunit (chains A, B, and C corresponding to COX I, COX II, and COX III, respectively), which are the larger subunits from complex IV (514, 227, and 261 residues, respectively). Thus, reliable random distribution of Σd*N* was generated. To this end, the codons from multiple sequence alignments of each chain were randomly sorted to form a subset of the same size as the original Exposed Noncontact subset of the corresponding chain. Afterwards, Σd*N* was computed using this random subset as explained above. For each chain, the random resampling was performed 10^4^ times to build up empirical distributions, which were used to contrast the Σd*N* values computed in the real Exposed Noncontact subsets.

### Fixed-Sites Codon-Substitution Models

The nonsynonymous/synonymous substitution rate ratio, ω, provides a measure of selective pressure at the amino acid level. As described above, in the Codon Sorting section, we have used structural information to partition sites into classes, which are expected to have different selective pressures and thus different ω ratios. Therefore, using such structural information we proceeded to fit models that assign different ω ratios for the different site partitions (Yang and Swanson 2002). To this end, we employed the codeml program, taking advantage of the G option of the sequence data file. Because of the large number of species being analyzed, we fixed the branch lengths (Yang 2000). The branch length provided by the program “dnaml” from PHYLIP package is defined as the expected number of nucleotide substitution per nucleotide site. However, we needed branch lengths expressed as the expected number of nucleotide substitutions per codon. Therefore, we used the codon model M0 to get branch lengths, afterwards other NSsites were run, with those branch lengths fixed.

### Thermodynamic Stability Changes

The thermodynamic stability changes, ΔΔG, of mutations were computed using the protein design tool FoldX version 3.0 (Guerois et al. 2002; Schymkowitz et al. 2005). FoldX uses a full atomic description of the structure of the protein, to provide a quantitative estimation of the importance of the interactions contributing to the stability of this protein. The three-dimensional (3D) structure of COX was subjected to an optimization procedure using the repair function of FoldX. Afterwards, an alanine scan was carried out, the resulting ΔΔG were recorded and used to calculate the means for Exposed Noncontact, Mt–mt Contact, and Mt–nu Contact residues.

### Molecular Visualization

**Fig. 2.—:**
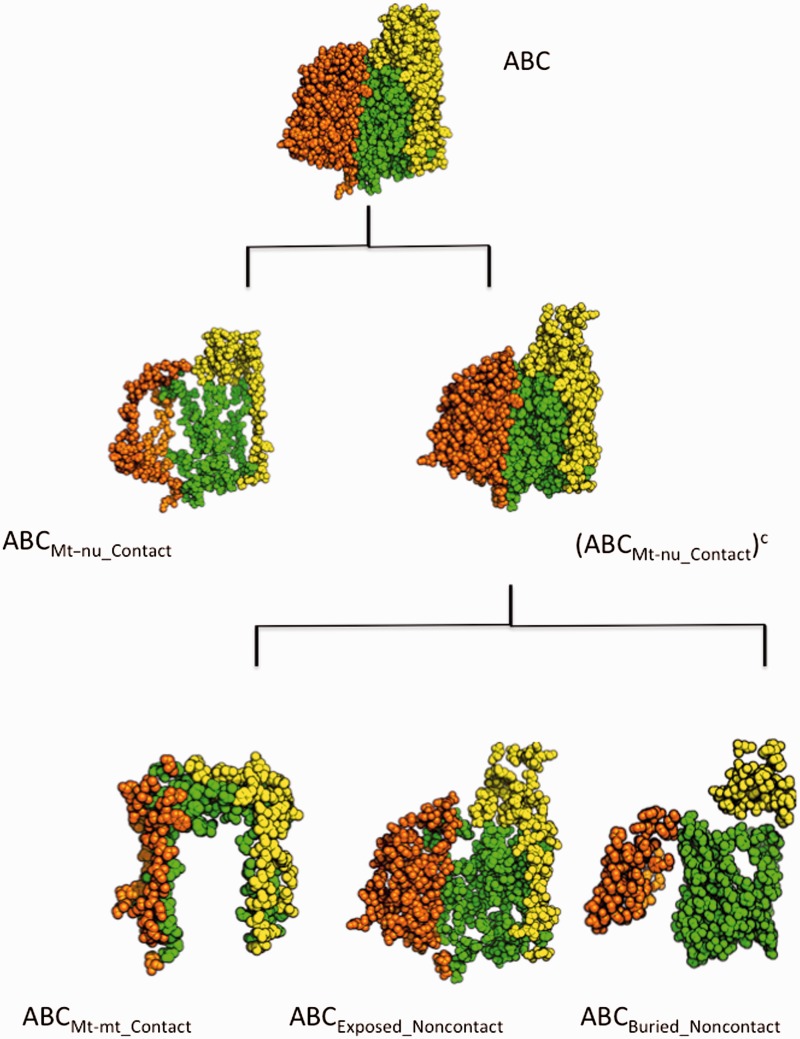
Structural view of mitochondrially encoded COX residues. COX core, consisting of COX subunits I (chain A in green), II (chain B in yellow), and III (chain C in orange) is shown at the top of the figure. The spatial distribution of those residues in close contact with nDNA-encoded subunits (ABC_Mt-nu_Contact_) is also shown. The set formed by mtDNA-encoded residues that are not in contact with nDNA-encoded subunits, (ABC_Mt-nu_Contact_)^c^, was partitioned into three disjoint subsets: ABC_Mt-mt_Contact_, which is formed by those residues contacting only with other mtDNA-encoded residues; ABC_Exposed_Noncontact_, encompassing residues accessible to the solvent that are not involved in intersubunit contacts; and ABC_Buried_Noncontact_, which contains all those residues that being buried inside the protein are not available for intersubunit contacts. The spatial distributions of the residues belonging to each of these subsets are shown at the bottom of the figure.

The structures shown in figures 2 and 7 were rendered using PyMol (http://www.pymol.org).

## Results and Discussion

### Discerning the Effects of Residue Interaction on the Evolutionary Rate from Other Structural Effects

The optimizing interaction hypothesis arose from the observation that mtDNA-encoded residues from COX in close contact with nDNA-encoded amino acids showed a higher rate of nonsynonymous substitutions than the rest of mtDNA-encoded residues. As these rates of nonsynonymous substitutions were originally calculated using orthologous sequences from only 26 mammalian species, we wanted to start by reassessing this issue using a more comprehensive collection of sequences from 371 mammalian taxa. In this way, using our extensive alignment and the methodology described by Schmidt et al. (2001), the set formed by the aligned codons from the three mtDNA-encoded COX subunits (ABC) was split into two subsets: ABC_Mt__–__nu_Contact_ and its complement, (ABC_Mt__–__nu_Contact_)^c^. The former encompassed those triplets encoding for residues close to nDNA-encoded amino acids in the holoenzyme, whereas the latter set contained all the codons from chain A (COX I), B (COX II), and C (COX III) that encoded amino acids that are not in contact with nDNA-encoded residues (fig. 2). The calculated interaction ratio (see Materials and Methods for definition), *R*_ABCMt__–__nu_Contact,(ABCMt__–__nu_Contact)_^c^, was 1.805, greater than 1. Although this result is in line with the data reported by Schmidt et al. 2001, such an observation by itself is, in our opinion, insufficient to support the conclusion that contact residues are subjected to a positive selection, as suggested in previous works.

In this sense, a number of considerations need to be addressed before any conclusion can be reached. For instance, the set formed by mtDNA-encoded residues that are not in contact with nDNA-encoded amino acids, ${\left({\text{ABC}}_{\text{Mt-nu\_Contact}}\right)}^{\text{c}}$, used as reference to compute the interaction ratio, represents a heterogeneous collection of residues. Thus, although the amino acids belonging to the ABC_Mt__–__nu_Contact_ group are mainly located at the protein surface, a significant part of the noncontact residues are buried into the protein structure (fig. 2). This observation is relevant because we have recently reported that for mtDNA-encoded proteins, buried residues are most likely to remain conserved during evolution compared with their solvent exposed counterparts (Aledo et al. 2012). Therefore, if we want to address the effects of residue interaction on the evolutionary rate, and discern them from other structural effects such as solvent exposure, the noncontact set used as reference should be restricted to avoid buried residues.

Although the above-mentioned restriction is necessary, it is not sufficient to build a suitable reference set. Indeed, the resulting set of such a restriction still contains a group of residues that may bias the results and mislead the conclusions. We are referring to the collection formed by those amino acids implicated in protein–protein interactions involving only contacts between mtDNA-encoded residues (see Mt–mt Contact in fig. 2). As the evolvability of this category of residues has not been previously characterized, we excluded them from the reference set, which finally was formed only by those mtDNA-encoded residues exposed at the protein surface that are not involved in any sort of intersubunit contacts. This reference set is referred to as Exposed Noncontact (fig. 2).

Once the partition of the initial data set had been carried out as described above and illustrated in figure 2, we next computed diverse evolutionary variables to characterize the relative evolvability of these different subsets of residues, all of them containing only codons for amino acids exposed at the protein surface. The results of such analyses are described next.

### The Exposed Noncontact Residues from COX I Are Exceptionally Conserved

**Fig. 3.—:**
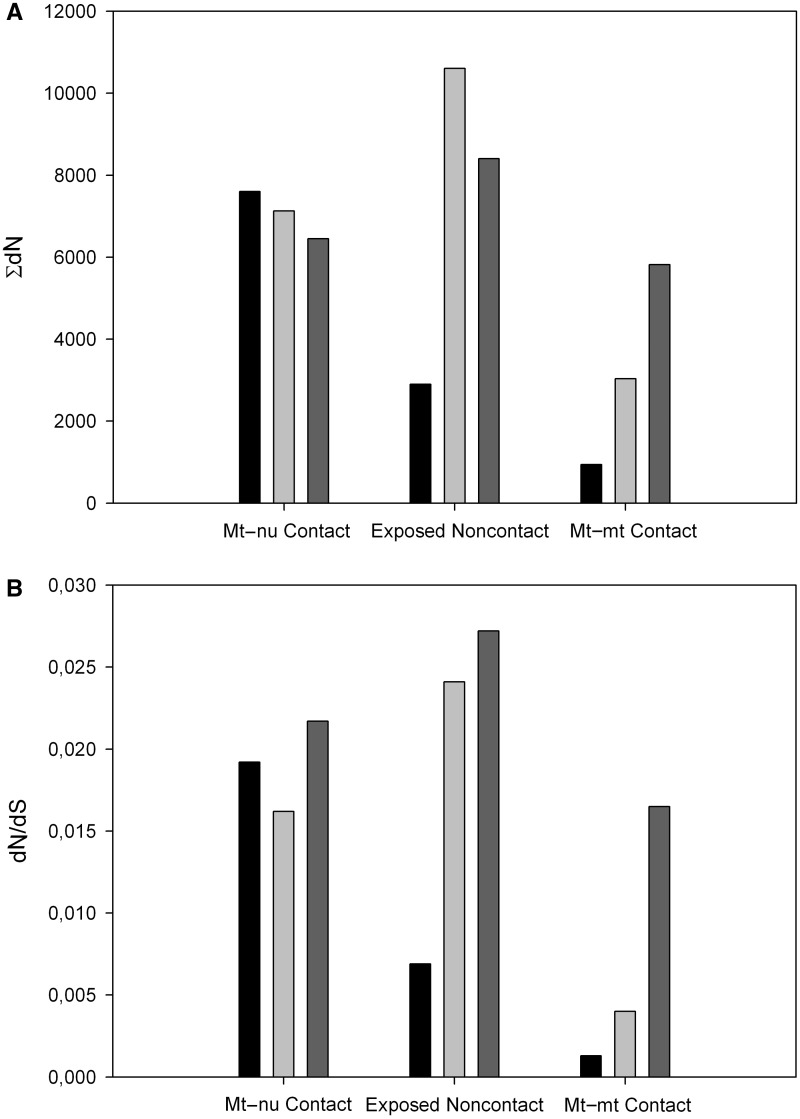
Exposed noncontact residues from COX I are conspicuously conserved. The categories of surface residues (Mt–nu Contact, Exposed Noncontact, and Mt–mt Contact) from each mtDNA-encoded chain were used to compute the corresponding Σd*N* and ω values, which are plotted in (*A*) and (*B*), respectively. Black, light gray, and dark gray bars represent COX I, II, and III, respectively. From this figure, it is evident that Exposed Noncontact residues from COX I behave uniquely, exhibiting little tendency to mutate. The standard errors, which are omitted from the figure, are without exception below 2.5%.

We recalculated the interaction ratio for the Mt–nu Contact category, but now using the Exposed Noncontact as the reference set. In this way, we obtained a ratio of 1.18, which although much lower than 1.81 (the value obtained when no proper control was used), it is still over the unit. At this point, one may feel tempted to conclude that Mt–nu Contact residues evolve almost 1.2 times faster than noncontact residues, which would favor the optimizing hypothesis. However, when each individual chain was separately analyzed, we reached a different conclusion. For instance, when the same Σd*N* values used to compute the interaction ratio were plotted for each subunit (fig. 3*A*), it becomes clear-cut that, with the exception of COX I, the Exposed Noncontact groups accumulate more nonsynonymous substitutions than their Mt–nu Contact counterparts. As Mt–nu Contact amino acids exhibit on average higher accessible surface areas than Exposed Noncontact residues (79.2 ± 43.6 vs. 56.2 ± 42.8 Å^2^, respectively), we can rule out the possibility that the former may be evolving slowly because they are more buried than Exposed Noncontact residues.

As the number of nonsynonymous substitutions per nonsynonymous site, d*N*, is informative of the combined effect of mutation and selection, we used Σd*N* (see Materials and Methods) as a proxy of the evolvability of the corresponding subset of residues. However, Σd*N* averages over all the analyzed mammalian species and, therefore, it provides little information on whether the evolutionary behavior summarized in figure 3*A* has broad phylogenetic distribution and is present in most mammalian lineages. To overcome this inconvenience, we reconstructed the phylogenetic tree for the 371 mammalian species being analyzed and implemented maximum-likelihood models for our prepartitioned data sets. These models, referred to as fixed-sites models (Yang and Swanson 2002), account for heterogeneous selective pressures among residues subsets by using different ω parameters for the partitions. Figure 3*B* shows the fitted values of ω for each residue category and subunit. As it can be observed, the results obtained using this phylogenetic approach are in line with those derived from pairwise comparisons, in the sense that, again with the exception of COX I, purifying selection seems to be more efficient among Mt–nu Contact residues with respect to the Exposed Noncontact group. These observations question the optimizing hypothesis, which is based on the idea that Mt–nu Contact residues evolve more rapidly than the other mtDNA-encoded residues, a condition that is only fulfilled by COX I (fig. 3). Does it mean that Mt–nu Contact residues from COX I are subjected to higher evolutionary rates to promote mitonuclear coevolution? In other words, is the optimizing hypothesis at least valid for COX I? As we will argue next, the answer to these questions must be negative.

If adaptive selection on COX I Mt–nu Contact residues were behind the observed departure of this subunit from the general trend, then one would expect similar constraints among the Exposed Noncontact residues from the three mtDNA-encoded COX subunits. However, the results shown in figure 3 suggest that the other way around may be the case. That is, although Mt–nu Contact residues, regardless of the subunit being analyzed, showed similar selective pressures as assessed by ω, the Exposed Noncontact residues from COX I seem to be subjected to a much stronger purifying selection when compared with COX II and COX III subunits (fig. 3).

**Fig. 4.—:**
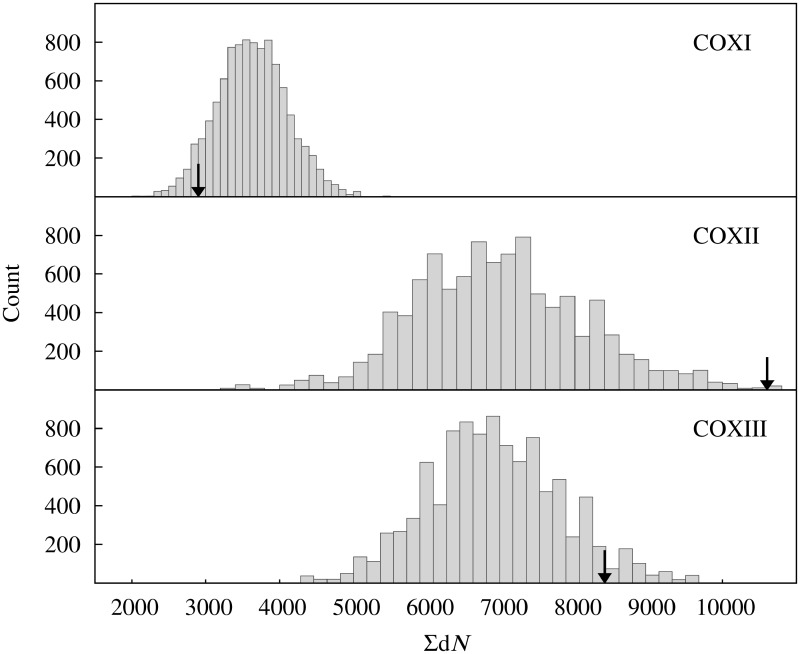
The behavior of Exposed Noncontact residues from COX I diverges from those exhibited by their counterparts in COX II and COX III. For each mtDNA-encoded chain, the codons from the multiple sequence alignment were randomly sorted to form a subset of the same size as the original Exposed Noncontact subset of the corresponding chain. Afterwards, Σd*N* was computed using this random subset. For each chain, the random resampling was performed 10^4^ times to build up empirical distributions, which were used to contrast the Σd*N* values computed in the real Exposed Noncontact subsets, which are indicated by arrows on the abscissa axis.

To provide statistical support to the conclusion that Exposed Noncontact residues from COX I are exceptionally conserved, while avoiding assumptions about the underlying distributions, we resorted to a bootstrap approach. Briefly, for each mtDNA-encoded chain, the codons from the multiple sequence alignment were randomly sorted to form a subset of the same size as the original Exposed Noncontact subset of the corresponding chain. Afterwards, Σd*N* was computed using this random subset. For each chain, the random resampling was performed 10^4^ times to build up empirical distributions, which were used to contrast the Σd*N* values computed in the real Exposed Noncontact subsets. As it can be deduced from figure 4, those residues belonging to the Exposed Noncontact groups from COX II and COX III are among the most variable residues (*P* values 0.003 and 0.045, respectively). In contrast, exposed noncontact residues from COX I were among the most conserved residues (fig. 4).

**Fig. 5.—:**
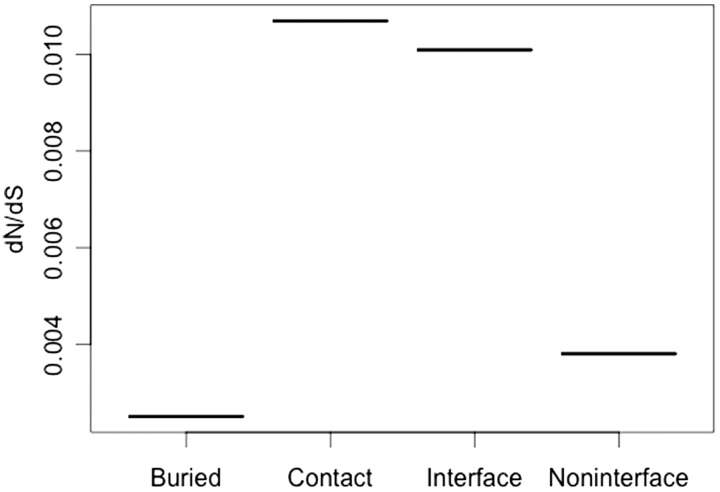
Uneven selective pressure on different COX I subset of residues. Using fixed-sites codon-substitution models, the selective pressure of the indicated subsets of residues was estimated. As it can be observed, residues from the COX I Exposed Noncontact group that do not take part in protein–protein interfaces are subjected to a remarkable selective pressure. The standard errors, which are omitted from the figure, are without exception below 2.5%.

As COX I, a polypeptide belonging to the core of the enzymatic complex, interacts with many other subunits from the complex, we reasoned that many residues from its surface may be involved in forming protein–protein interfaces, even when they would not establish interatomic contacts. Therefore, we addressed whether those residues taking part of such interfaces may be responsible for the unusual high degree of conservation described above. To this end, the COX I Exposed Noncontact set (143 residues) was partitioned into “Interface” (68 residues) and “Noninterface” (75 residues) subsets, according to the criterion described in Materials and Methods. Briefly, the residue being sorted was considered as Interface if its surface area was reduced when considered in the complex with respect to the single subunit, otherwise the amino acid was classified as Noninterface*.* This partition, together with the phylogenetic tree that we had obtained, allowed us to use the program codeml from the PAML package to assess the selective pressure suffered by the different residue categories. The results of such analyses were clear and intriguing (fig. 5). Those solvent-exposed residues from COX I that are neither engaged in intersubunit contacts nor involved in protein interfaces suffer a surprisingly high selective pressure, as judged by a low ω value comparable with that estimated for buried residues, but significantly lower (*P* < 0.05) than the ω value found for the Interface group (fig. 5). In contrast, those Exposed Noncontact residues from COX I that were involved in protein–protein interfaces showed a much higher ω value, similar to that obtained for contact residues.

**Fig. 6.—:**
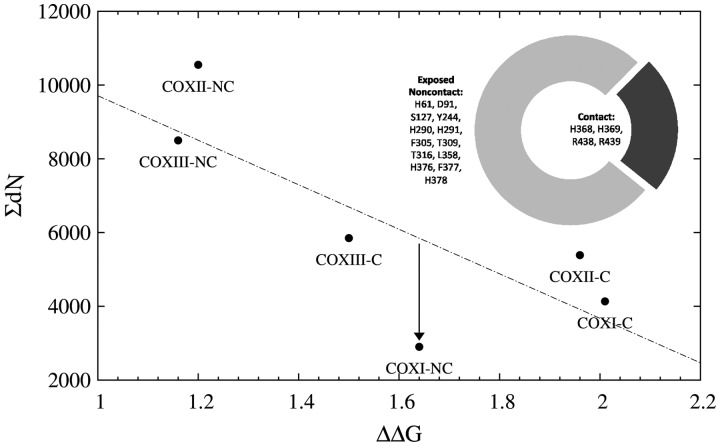
Exposed Noncontact residues from COX I are much more conserved than expected from their destabilizing effect on the holoenzyme. The exposed residues from each mtDNA-encoded proteins were split into two groups: Contact (C) and noncontact (NC), according to the criteria given in the text. Afterwards, the Σd*N* and mean ΔΔG were computed for each of these subsets. These two variables showed a significant negative correlation (*n* = 6, Pearson *r* = −0.774, *P* value = 0.035) that was improved when data corresponding to COX I Exposed Noncontact were excluded from the analysis (*n* = 5, Pearson *r* = −0.890, *P* value = 0.021). The inset shows the proportion of residues that have been described as functionally relevant among the Exposed Noncontact, in comparison with the proportion of these residues that belong to the Contact group.

In an attempt to get further insight into the particular forces imposing such exceptionally high degree of conservation among Exposed Noncontact COX I residues (particularly within the Noninterface subset), we assessed the effect of in silico alanine scanning mutagenesis on the stability of the complex. Although the mean ΔΔG for Exposed Noncontact COX I residues (1.64 kJ/mol) was significantly higher than those values from COX II (1.20 kJ/mol) and COX III (1.16 kJ/mol), *P* values 0.015 and 0.008, respectively, the contribution of COX I Exposed Noncontact residues to the whole thermodynamic stability of the holoenzyme can hardly be invoked as a reason for their high degree of conservation, as it is evident from figure 6. For each mtDNA-encoded protein, the Contact and Exposed Noncontact subsets were employed to compute their Σd*N* values, which were plotted against their corresponding ΔΔG mean values (fig. 6). We found a significant negative correlation between these two variables (*P* value = 0.035), indicating that those substitutions that tend to be more destabilizing are also more constrained, which is in line with our previous observations that thermodynamic stability plays a relevant role in the evolvability of mtDNA-encoded proteins (Aledo et al. 2012). However, the COX I Exposed Noncontact group showed up as an outlier. In this sense, for a ΔΔG of 1.64 kJ/mol (the mean value computed for COX I Exposed Noncontact residues) the expected Σd*N* should be higher than twice the observed Σd*N* (fig. 6). In other words, whatever the constraining forces may be, they seem to be unrelated to structural stability.

During the past decade, significant experimental evidence supports the organization of the mitochondrial respiratory chain into higher order structures known as respirasome (Schagger and Pfeiffer 2000; Acin-Perez et al. 2008; Winge 2012). We wondered whether Exposed Noncontact residues from COX I might be involved in functions related to the formation of mitochondrial supercomplexes, providing in this way an explanation for the intriguing high degree of conservation of this subset of residues. Along the same lines, two quasi-simultaneous recent studies have reported the 3D structure of mitochondrial supercomplex I_1_III_2_IV_1_, determined by electron cryomicroscopy at 19–22 Å resolution (Althoff et al. 2011; Dudkina et al. 2011). Fitting of X-ray structures of single complexes I, III_2_, and IV with high fidelity unravels only a few sites where neighboring complexes come close enough for ion bridges or hydrogen bonds. Three of such sites of potentially strong protein–protein interaction were found between complexes III and IV. However, none of these sites was on COX I (Althoff et al. 2011; Dudkina et al. 2011). Nevertheless, beside the supercomplex I_1_III_2_IV_1_, other forms of supercomplexes such as I_1_III_2_IV_2_, I_1_III_2_IV_3_, and I_1_III_2_IV_4_ have been described in bovine heart mitochondria (Schagger and Pfeiffer 2001). As the three-dimensional structures of these less abundant forms of supercomplexes are unknown, the involvement of residues from COX I in the assembly/stability of respirasomes, although unlikely, cannot be completely ruled out.

We next explored other alternatives. In this sense, it is widely acknowledged that functional regions of proteins exhibit higher inertia to nonsynonymous changes than those other regions (Glaser et al. 2003). Hence, if there were a higher proportion of functionally important residues in the Exposed Noncontact region of COX I than in its contact counterpart, this would help to explain the extraordinarily low Σd*N* and ω computed for the set Exposed Noncontact from COX I. To test this possibility, we focused on those COX I residues that have been described to fulfill important functions such as the binding of heme, Cu and Mg, proton pumping, and electron transfer (Michel et al. 1998; Yoshikawa et al. 1998). Figure 6 inset shows that the proportion of these residues that belong to the Exposed Noncontact set override the proportion of those that pertain to the *Contact* group. Although this observation is qualitatively in line with the very low Σd*N* and ω observed for the COX I Exposed Noncontact ensemble, it should be noted that the catalog of functional residues we have used is very limited in size and probably it is far from the complete inventory, which hampers obtaining quantitative statistical support. Nevertheless, the exceptionally high degree of conservation observed among Exposed Noncontact COX I residues likely captures important constraints that apply to biological functionality yet to be unraveled. Therefore, we proceeded to examine other hypotheses related to the structure–function relationship of this set of exceptionally conserved residues.

To this end, we turned the focus toward the O_2_ reduction environment of the enzyme. Indeed, beside those residues that have been described as being directly involved in binding the prosthetic groups, side chains from other residues in the vicinity may influence the rate of oxygen reduction to water. For instance, heme-pocket differences between the α and β hemoglobin subunits have been pointed as determinants of the relative affinities for O_2_ and other ligands (Markovska et al. 1975). More generally, data gathered from studies with different iron porphyrin-containing proteins suggest that structural factors can modulate the reduction potential of the bound heme by stabilizing or destabilizing the ferric and ferrous heme proteins (Reedy et al. 2008). Thus, we hypothesized that the highly conserved residues from the COX I Exposed Noninterface set may contribute to configurate a suitable environment for the redox reactions that this protein catalyzes.

**Fig. 7.—:**
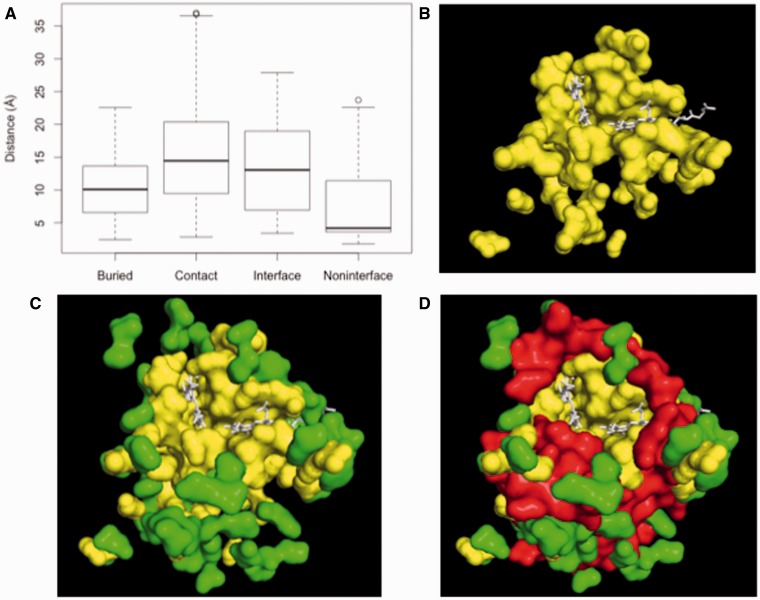
The physical proximity to the heme groups may be a strong determinant of the selective pressure. The distance in angstroms of each COX I residue to the closest heme group was determined and the distribution of such a variable is shown for each category of residue (*A*). (*B*) shows, in yellow, those Exposed Noncontact residues that are not involved in the formation of interfaces, as well as their spatial proximity to the hemes *a* and *a3*, in white. In (*C*), it has been added, in green color, those Exposed Noncontact residues that form part of protein–protein interfaces. Finally, for comparative purposes, in (*D*) the Buried residues are shown in red.

As a first approach to test this hypothesis, we computed the distance of each COX I residue to the closest heme group. The distribution of these distances within each residue category is presented in figure 7*A*. The comparison of figures 5 and 7*A* suggests that the physical proximity to the heme groups may be a strong determinant of the selective pressure. In any case, there is no doubt that the solvent-exposed residues from COX I that are neither engaged in intersubunit contacts nor involved in protein interfaces are among the amino acids closest to the heme groups, as well as among the residues with lower ω value. The closeness of *Noninterface* residues to the prosthetic groups was further confirmed by visual inspection of the structure (fig. 7*B* and *C*). All together, our data suggest that this set of residues is under a high selective pressure to keep a suitable redox environment around the heme prosthetic groups. In any event, the high degree of conservation among the Exposed Noncontact residues from COX I herein described is a remarkable observation that must be taken into account when addressing evolutionary issues related to the COX complex, otherwise deceiving conclusions may be reached.

### Differential Behavior between Mt–mt and Mt–nu Interactions

As discussed above, those mtDNA-encoded residues at the protein surface that are not involved in intersubunit contacts are less constrained than those amino acids that form bounds with nDNA-encoded subunits (Mt–nu residues), with the exception of COX I. This conclusion is in line with the expectation that contact residues are in general much more likely to be under strong evolutionary constraint compared with noncontact residues. However, an obvious question that arises at this point is “how do Mt–nu residues compare with Mt–mt positions, in terms of evolvability?”

**Fig. 8.—:**
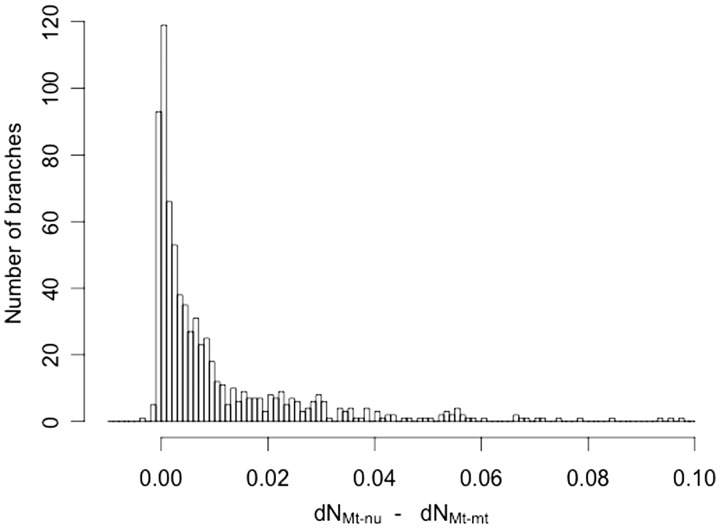
Residues involved in Mt–mt interactions are broadly conserved through phylogeny. The phylogenetic relationship among the 371 mammalian species of this study was reconstructed. The obtained tree was used to calculate the number of nonsynonymous substitutions per nonsynonymous site on a lineage-by-lineage basis, using the program “codeml” from the PAML package. In this way, within each category of codons, the d*N* was computed for each of the 739 branches. To assess whether the d*N* values within a codon category tend to be higher or lower than the d*N* values computed for other codon category, we proceeded in the following way. For a given branch, the difference d*N*_Mt–nu_ − d*N*_Mt–mt_ was calculated. Afterwards, using data from all the branches, an abundance histogram was plotted.

As it can be observed in figure 3, among all exposed residues, those involved in interactions between different mtDNA-encoded chains were the most constrained, showing both the lowest ω and Σd*N* values, regardless of the mtDNA-encoded subunit being considered. This observation indicates a much stronger purifying selection among Mt–mt Contact residues than among Mt–nu Contact amino acids. To substantiate this conclusion, we next assessed whether this differential behavior of the two subsets of contact residues had a broad phylogenetic distribution and was present in most mammalian lineages. To this end, we used the reconstructed tree to apportion the COX I nonsynonymous substitutions for the two categories of contact sites. Figure 8 shows the distribution of the statistic d*N*_Mt__–__nu_ − d*N*_Mt__–__mt_ among the phylogenetic tree branches, which was clearly biased toward positive values. Overall, these results suggest that those mtDNA-encoded residues in contact with mtDNA-encoded residues from a different chain are subjected to stronger constraints than those involved in interactions with nDNA-encoded subunits. In addition, this behavior seems to have a broad phylogenetic distribution and is valid for most mammalian lineages. To the best of our knowledge, this is the first quantitative study supporting such a conclusion.

Once we had established that residues from the Mt–mt Contact group were much more constrained than those belonging to the Mt–nu Contact set, we wondered whether such differential pattern might have arisen from a differential contribution of these two categories of contacts to the stability of the complex. To address this issue, we carried out an in silico alanine scanning mutagenesis. The results of such analysis are summarized in table 1. As expected, changes affecting *Noncontact* residues were among the least destabilizing mutations. On the other hand, from a thermodynamic point of view, residues from the Mt–nu Contact class were much less tolerant to changes than any other kind of residue, including those from the Mt–mt Contact set, which exhibited an intermediate behavior. As mutations between residues from the Mt–mt Contact class tend to be less destabilizing than those affecting residues from the Mt–nu Contact group, the higher degree of conservation observed between Mt–mt Contact residues does not seem to be based on thermodynamic stability, suggesting that functional aspects may be behind the high degree of conservation observed among Mt–mt Contact residues.

**Table 1:** Thermodynamic Stability Changes

	ΔΔG (kJ/mol)			
	ABC	COX 1	COX 2	COX 3
Exposed Noncontact	1.41 ± 1.51^†,§§^	1.64 ± 1.69^§^	1.20 ± 1.09^†,§§^	1.16 ± 1.38^§^
Mt–mt Contact	1.78 ± 1.97*	1.98 ± 1.65	1.92 ± 2.61	1.06 ± 1.52^§^
Mt–nu Contact	1.92 ± 1.60**	2.03 ± 1.55*	1.99 ± 1.47**	1.69 ± 1.73*^,†^

Note.—The number of “Exposed Noncontact” residues from COX I–III were 144, 56, and 92, respectively. The number of “Mt–mt Contact” residues from COX I–III were 95, 54, and 37, respectively. The number of “Mt–nu Contact” residues from chain COX I–III were 120, 74, and 82, respectively. Data are expressed as mean ± standard error.

Significantly different from Exposed Noncontact: *P < 0.05, **P < 0.0005; significantly different from Mt–mt Contact: ^†^*P* < 0.05; significantly different from Mt–nu Contact: ^§^*P* < 0.05, ^§§^*P* < 0.0005.

Although unlikely, the higher ω values computed for Mt–nu residues, with respect to that observed for the Mt–mt set, may be compatible with positive selection among certain residues from the Mt–nu set. Alternatively, and more likely, these results are also consistent with a relaxation of functional constraints (purifying selection) within this Mt–nu category of residues. To rule out the first scenario, we carried out likelihood ratio tests of positive selection using the program codeml from the PAML package (Yang 2007). For this purpose, we compared M1a (Nearly Neutral) and M2a (Positive Selection) models (Wong et al. 2004). We failed to reject M1a in favor of M2a in all the cases (*P* value > 0.998). That is, we could not infer positive selection acting on any of the three mtDNA-encoded COX subunits. Thus, our results rather point to a relaxation of purifying selection acting on the Mt–nu positions, particularly when compared with Mt–mt residues. To rationalize this conclusion, we reasoned as follows.

In mammals, the three mtDNA-encoded subunits form the catalytic core of the enzyme (Yoshikawa et al. 2011). Prokaryote forms of COX boil down to the catalytic core, which seems to have an ancient origin (Castresana et al. 1994). In contrast, eukaryotes possess nuclear genes for additional subunits, the number of which generally increases with the organismal complexity. In mammals, these additional subunits are nDNA-encoded and are thought to act as a regulatory shield surrounding the core (Soto et al. 2012). Although neither the origin nor the specific function of these additional noncatalytic subunits is completely understood, there is little doubt that they are significantly younger than the proteins conforming the catalytic core (Castresana et al. 1994; Das et al. 2004; Little et al. 2010). On the other hand, it is well known that young proteins tend to experience weaker purifying selection and evolve more quickly than old proteins (Alba and Castresana 2005; Vishnoi et al. 2010). Herein, we propose that what is true for proteins may also be true for interactions. In other words, it is reasonable to assume that within a given protein with a fixed antiquity, those residues involved in “old interactions” evolve more slowly than those other residues implicated in “young interactions.”

Describing how selection pressure acts at the interfaces of protein–protein complexes is a fundamental issue with high interest for the structural prediction of macromolecular assemblies. Therefore, although it is out of the scope of the current research, in the future it would be interesting to assess whether the relationship between the strength of selection and the age of the interaction, described herein for mtDNA-encoded COX subunits, also applies to other proteins.

## Concluding Remarks

Interactions of mtDNA- and nDNA-encoded proteins provide unique opportunities to study the evolution of protein–protein interactions and the effects of these interactions on the evolution of their respective genomes. To this respect, mammalian COX complex is well suited for studying the evolution of within- and between-genome interactions because a functional complex requires three mtDNA-encoded and ten nDNA-encoded subunits, accounting for a large number of interacting residues. Previous studies carried out with this complex have suggested that mtDNA-encoded residues in close physical contact with nDNA-encoded amino acids may be subjected to positive selection to optimize the structural–functional interaction between subunits from different genetic origins. This conclusion was based on the higher rate of nonsynonymous substitutions observed among mtDNA-encoded residues in contact with nDNA-encoded residues, with respect to the rest of mtDNA-encoded amino acids. However, as we have shown herein, failing to discern the effect of interaction from other confounding factors can lead to misleading conclusions. Thus, when such corrections were made, data analysis showed that mtDNA-encoded residues engaged in contacts are, in general, more constrained than their noncontact counterparts. Nevertheless, noncontact residues from the surface of COX I subunit are a remarkable exception, being subjected to an exceptionally high purifying selection, that may be related to the maintenance of a suitable environment for the catalytic reduction of oxygen to water.

Besides disclosing the remarkable conservation of the COX I nonbinding surface, and providing compelling evidence against the so-called optimizing interaction hypothesis, our approach also allowed to make some interesting findings. For instance, those mtDNA-encoded residues in contact with mtDNA-encoded residues from a different chain are subjected to stronger constraints than those involved in interactions with nDNA-encoded subunits. This observation cannot be explained on the basis of thermodynamic stability, because interactions between mtDNA-encoded subunits contribute more weakly to the complex stability than those interactions between subunits encoded by different genomes. Therefore, the higher conservation observed among mtDNA-encoded residues involved in within genome interactions is likely due to factors other than structural stability.

## Supplementary Material

Supplementary materials S1–S3 are available at *Genome Biology and Evolution* online (http://www.gbe.oxfordjournals.org/).

Supplementary Data
