# Supplementary material for: Protein–Protein Interfaces from Cytochrome c Oxidase I Evolve Faster than Nonbinding Surfaces, yet Negative Selection Is the Driving Force
Source: Genome Biol Evol. 2014 Oct 29;6(11):3064–76. doi: 10.1093/gbe/evu240 (PMC4255772; doi:10.1093/gbe/evu240)
Supplement: Supplementary Data [file supp_6_11_3064__index.html]

Protein-protein interfaces from cytochrome c oxidase I evolve faster than nonbinding surfaces, yet negative selection is the driving force — Protein–Protein Interfaces from Cytochrome c Oxidase I Evolve Faster than Nonbinding Surfaces, yet Negative Selection Is the Driving Force — Supplementary Data 

# Protein–Protein Interfaces from Cytochrome c Oxidase I Evolve Faster than Nonbinding Surfaces, yet Negative Selection Is the Driving Force

## Supplementary Data

files

**Files in this Data Supplement:**

- Supplementary Data - zip file
